# Sarcoid-Like Mediastinal Lymphadenopathy in Gynecologic Malignancy

**DOI:** 10.1155/2018/5141575

**Published:** 2018-02-14

**Authors:** Bilal H. Lashari, Megumi Asai, Gissele Randleman, Martha Sack, Rajeshkumar Patel

**Affiliations:** ^1^Department of Internal Medicine, Abington Jefferson Health, Abington, PA, USA; ^2^Department of Surgery, Abington Jefferson Health, Abington, PA, USA; ^3^Department of Pathology, Abington Jefferson Health, Abington, PA, USA; ^4^Pulmonary-Critical Care Medicine, Abington Jefferson Health, Abington, PA, USA

## Abstract

Noncaseating granulomas are seen surrounding tumors with varying frequency, possibly as part of an immune response to tumor cells. However, data about the association of sarcoid with gynecologic malignancy is sparse. We performed a search of our institutional database for all EBUS-TBNA biopsies conducted within the past five years that revealed granulomatous inflammation. All adult female patients with a history of gynecologic malignancy were included. Patients with a history of sarcoidosis or fungal or mycobacterial infection were excluded. All patients with evidence of malignant cells on TBNA specimen were excluded. Our results revealed 65 patients with histologic diagnosis of a noncaseating granuloma on EBUS-TBNA. Five patients (7.69%) had a history of gynecologic malignancy. Two patients had evidence of PET-positive nodes on surveillance scans, which led directly to the examination. Our findings suggest that distant malignancies may cause granulomatous lymphadenitis, through yet undefined mechanisms. As such, patients with evidence of mediastinal lymphadenopathy could benefit from routine sampling and histologic examination to define the pathology in the correct clinical context.

## 1. Introduction

Granulomatous inflammation in lymph nodes can be attributable to nonspecific inflammation such as sarcoidosis or secondary to fungal and mycobacterial infection or foreign body reaction to respiratory inhalants [1]. It has been associated with neoplastic disease in regional and distant lymph nodes. It was described in association with Non-Small Cell Lung Cancer [2] and as high as 14% in Hodgkin disease [3]. The causes of this phenomenon are currently unknown.

Recent studies have indicated that granulomatous lymphadenitis may precede the development of and follow the resolution of a wide range of malignancy [4, 5].

Regional chest diseases and lymphoma understandably involve mediastinal and hilar lymph nodes. However, little data is available regarding the relationship of nonlocal malignancy and mediastinal lymphadenopathy, especially gynecologic malignancy [6].

## 2. Materials and Methods

After IRB approval (IRB Number 16-046) a retrospective review was performed of all endobronchial ultrasound guided transbronchial needle aspiration (EBUS-TBNA) biopsies performed between 2013 and 2016 at our institution, with histologic findings of granuloma, histiocytes, and giant cells. All female patients with age greater than 18 were included. Patients with a history of or other clinical features consistent with sarcoidosis or evidence of mycobacterial or fungal infection by tissue staining or culture were excluded. In addition, all patients with evidence of malignant cells on tissue sampling, in addition to granulomatous features, were excluded.

## 3. Procedure

All bronchoscopy procedures were performed under general anesthesia. A comprehensive and systematic ultrasonic examination of the mediastinum and hilar lymph nodes was conducted followed by transbronchial needle aspiration of lymph nodes >10 mm in short axis diameter. At least five passes were made per node.

## 4. Results

Our search yielded 65 patients with pathological diagnosis of noncaseating granuloma. Tissue staining for acid fast organisms and fungi were negative in all patients. Pathological analysis of EBUS-TBNA samples did not identify malignant cells.

Five patients had a history of gynecologic malignancy. The malignancies and characteristics of these patients are described in Table 1. All five were found to have mediastinal lymphadenopathy on surveillance CT scans as part of regular follow-up.

Two patients had a history of serous ovarian carcinoma and FIGO stages IIIA and IIIB at diagnosis, underwent appropriate therapy, and had been disease-free for two years. Of these, one patient had PET-positive nodes in right hila, with increased metabolic activity with an SUV max of 3.7; this patient had stage IIIB disease and had been treated with IV Taxol and Intraperitoneal Cisplatin and Taxol after undergoing TLH and BSO. The second patient did not have a PET scan; she had stage IIIA disease at diagnosis, two years before the diagnosis of mediastinal lymphadenopathy and had successfully undergone TAH-BSO and subsequent chemotherapy with Taxol-Cisplatin IV, neither patient had any documented mediastinal lymphadenopathy on initial staging CT scan.

One patient had FIGO IA Endometrial Adenocarcinoma diagnosed a year prior to PET-positive bilateral hilar lymphadenopathy. PET was remarkable for bilateral hilar uptake, with SUV max in representative left hilar nodes at 6.6, infracarinal node at 6.9, and right hilar node at 6.2. This patient had bilateral lymphadenopathy evident on initial staging CT, and sampling was performed for staging purposes. She had undergone TLH and BSO and was not recommended chemotherapy based on the localized nature of disease.

Another patient had a remote history of FIGO IC Endometrial Adenocarcinoma and was found to have right hilar lymphadenopathy on CT imaging, without PET scan four years after completion of treatment. Initial staging CT was negative for hilar lymphadenopathy, and chemotherapy regimen for this patient was unavailable.

The last patient had a remote serous fallopian carcinoma and FIGO IIIC and subsequently developed mediastinal lymphadenopathy found on CT. PET scan and chemotherapy regimen was not available for this patient.

No patient had radiologic evidence of parenchymal lung disease during their evaluation. All patients remained clinically and radiographically stable from a respiratory standpoint.

## 5. Discussion

A sarcoid-like reaction is caused by a multitude of reasons including but not limited to infection (fungal, mycobacterial), autoimmune disorders, interstitial lung disease, chemotherapy, and chemical agents. Histology in granulomatous lymphadenitis is characterized by scattered small epithelioid granulomas with a sparse arrangement of epithelioid cells and accompanying lymphocytic infiltrate among granuloma cells [7]. Sarcoid-like reaction as a tumor-related response was first described in 1917 by Herxheimer, in five patients [8]. It has been reported as frequently as 4-5% of all carcinomas. Approximately 14% of patients with Hodgkin disease and 7.3% of Non-Hodgkin lymphoma have sarcoid-like lymphadenopathy. In one report almost 50% of patients with Seminoma have this finding [9]. In most malignancies, the granulomatous reaction is seen near the tumor cells [9]. There are case reports of distant nonmetastatic granulomatous lymphadenopathy in association with lung and gastrointestinal malignancies [10] and a range of solid tumors, by Grados et al. [11]. Our case series of granulomatous mediastinal lymphadenitis at a site remote from the location of primary gynecological malignancy without evidence of metastatic disease is unique in that sense.

It has been postulated that granuloma formation may play a role in host defense against metastasis [9]. Development of reactive granulomas in local lymph node groups draining the area occurs as tumor antigens are exposed to T cells after necrosis or tumor cell lysis. This can explain regional lymphadenitis but cannot explain the same phenomenon in remote lymph node groups. Similarly, systemic stimulation of T cell immunity by tumor antigen may explain granulomatous reaction at a site distant from malignancy but would not describe localized mediastinal lymphadenopathy. DePew et al. published a case series of 1275 patients of which 154 were found to have granulomatous lymphadenitis and revealed a history of malignant disease in 7.7% of patients [12]. While the pathogenic mechanism of these findings remains unclear, it has been demonstrated that the presence of granuloma does not positively or adversely affect outcome in cancer patients [13]; it is, however, important to note that patients with sarcoidosis have an increased risk of developing a malignancy [14].

The exact mechanism of our observation is unknown. One simple reason could be a T cell response to a tumor antigen, and if this is the case, it should be seen in most patients with cancer. Also, patients may have developed these reactions to chemotherapy, but this does not explain the localized occurrence in mediastinal and hilar lymph nodes. Micrometastatic disease with local T cell response can explain our findings as well. Lastly, these patients may have developed granulomatous lymphadenitis entirely independent of the disease, diagnosed incidentally during the surveillance CT or PET scan.

Our results demonstrate the importance of multidisciplinary approach as one of the emerging tools in cancer management. Gynecological oncology was introduced to the endobronchial ultrasound (EBUS) guided sampling of mediastinal and hilar lymph nodes, a tool completely foreign to this specialty [15]. EBUS-TBNA is now the standard of care in sampling lymph nodes for staging of small cell lung cancer [16]; it can also become a helpful tool for nonpulmonary malignancy.

## 6. Conclusion

Mediastinal and hilar granulomatous lymphadenitis can be seen in patients with gynecological malignancy without metastatic disease. The exact pathologic mechanisms for the development of this reaction are unclear. PET and CT scan can give a falsely positive impression of metastatic disease. EBUS guided sampling of mediastinal and hilar lymph nodes can be a useful tool in the evaluation of patients with mediastinal lymphadenopathy to confirm or refute metastatic disease even in gynecological malignancy.

## Figures and Tables

**Table 1:** 

Age	Primary malignancy	FIGO stage at diagnosis	Sampled LN station	PET scan	PET uptake	Initial chemotherapy	Lymphadenopathy on initial staging CT scan	Duration in which patient was followed after EBUS and remained disease-free
66	Endometrial Adenocarcinoma	IA	4R, 7, 12L	Positive	L hilum maximal SUV of 6.6. Infracarinal maximal SUV of 6.9. R hilum maximal SUV of 6.2	No chemotherapy advised	Mediastinal lymphadenopathy present on initial staging CT	2 years

68	Serous ovarian carcinoma	IIIB	7 and 11R	Positive	R hilar maximal SUV of 3.7	Taxol IV, Cisplatin intraperitoneal, and Taxol intraperitoneal	None	1 year

33	Serous ovarian carcinoma	IIIA	4R, 7, 11R	N/A	N/A	Taxol/Cisplatin chemotherapy	None	3 years

67	Serous fallopian carcinoma	IIIC	4R, 7, 10R	N/A	N/A	N/A	None	N/A

58	Endometrial Adenocarcinoma	IC	4R, 7, 11R	N/A	N/A	N/A	None	4 years
